# Physical-chemical characterization and bond strength to zirconia of dental adhesives with different monomer mixtures and photoinitiator systems light-activated with poly and monowave devices

**DOI:** 10.1080/26415275.2022.2064289

**Published:** 2022-04-28

**Authors:** Constantino Fernandes Neto, Mayara Hana Narimatsu, Pedro Henrique Magão, Reginaldo Mendonça da Costa, Carmem Silvia Pfeifer, Adilson Yoshio Furuse

**Affiliations:** a Department of Operative Dentistry, Endodontics and Dental Materials, Bauru School of Dentistry, University of São Paulo, Bauru, Brazil; b Division of Biomaterials and Biomechanics, Department of Restorative Dentistry, School of Dentistry, Oregon Health and Science University, Portland, OR, USA

**Keywords:** Adhesives, ceramics, zirconia, bond strength

## Abstract

**Introduction: **Bonding to crystalline zirconia is currently a challenge. Properly cured adhesives are crucial to optimize this bond, and that in turn is influenced by the initial mobility of the system, as well as by the reactivity of the initiators. **Aim:** This study aimed to characterize adhesives containing monomer mixtures of different viscosities and double and triple photoinitiator systems; and to evaluate the bonding to Y-TZP zirconia, when adhesives were light-activated with monowave or polywave light-curing units (LCU). **Materials and methods: **Adhesives were formulated at a 1:1 weight proportion of Bis-GMA/TEGDMA or Bis-GMA/Bis-EMA. To these mixtures 0.5 wt% of CQ, 0.5–1.0 wt% of DABE, 0.5–1.0 wt% of DPIHP, or 0.5–1.0 wt% of TAS-Sb were added and used as photoinitiator systems. A total of ten adhesives were prepared. Resin composite cylinders were cemented on zirconia slices and 6000 thermal cycles were performed. Degree of conversion (DC), sorption (SO) and solubility (SL) after 7 days of water storage, and microshear bond strength (µSBS) were evaluated. Data were analyzed with three-way ANOVA and Tukey’s HSD (*α* = 0.05). **Results:** Bis-GMA/Bis-EMA combined with either CQ/DABE or CQ/DABE/TAS-Sb presented the highest DC, and no significant differences were observed for LCUs (*p* = .298). CQ/DABE < CQ/DABE/TAS-Sb ≈ CQ/DABE/DPIHP and the polywave LCU showed smaller overall SO (*p* < .05). Bis-GMA/TEGDMA with CQ/DABE cured with the polywave LCU presented the lowest SO. SL varied as follows: CQ/DABE/TAS-Sb < CQ/DABE/DPIHP < CQ/DABE (*p* < .001). For µSBS, only the factor photoinitiator system was significant (*p* = .045). All mean values were above 30 MPa, with higher values being observed for BIS-GMA/TEGDMA and CQ/DABE. **Conclusion:** It can be concluded that the adhesive containing CQ/DABE/TAS-Sb as coinitiator of Bis-GMA/Bis-EMA mixtures produced a material with higher DC and lower SL, while bond strength values were similar to the ones obtained by CQ/DABE.

## Introduction

Yttria-stabilized tetragonal zirconia polycrystal (Y-TZP) is a polycrystalline ceramic with excellent physical, mechanical, and thermal properties, presenting high biocompatibility [1]. Although Y-TZP ceramics present all these excellent properties, due to the nature of its microstructure the efficacy of the adhesive cementation procedure is still questionable. In order to overcome this issue, different surface treatments have been suggested to increase reactivity and improve the bonding to the zirconia surface [2–5], of which air abrasion with particles such as silica-coated alumina particles has shown promising results by increasing mechanical interlocking and surface wettability [4]. However, of equal importance is a properly cured adhesive layer, which is influenced by the initial viscosity (mobility) of the system [6], as well as the reactivity of the initiator system. In addition, the light activation device needs to closely match the absorption spectrum of the initiator, as well as have sufficient light intensity. This issue is especially true considering the fact that the ceramic’s thickness and microstructure will certainly attenuate the light reaching the resin cement during the cementation.

Adhesives are mainly composed of monomer mixtures and a photoinitiator system [7]. Monomers contain methacrylates that are responsible for binding to the silane coupling agent and forming the resin matrix with monomers of the cement, while photoinitiators trigger the polymerization reaction. The monomer mixture and the photoinitiator of choice will influence the final performance [8–10]. Bisphenol A glycidyl dimethacrylate (Bis-GMA) is a highly reactive monomer present in most adhesive and resin composite formulations. However, the presence of hydroxyl groups in its backbone increases its viscosity and lowers its degree of conversion (DC) [11]. In turn, the viscosity of the monomers greatly influences polymerization rate, since it determines the early onset of diffusion limitations to propagation [6,12]. For this reason, less viscous co-polymers such as triethylene glycol dimethacrylate (TEGDMA), ethoxylated bisphenol methacrylate (Bis-EMA), urethane dimethacrylate (UDMA) and 2-hydroxyethyl methacrylate (HEMA) are added [9,13]. In this sense, Bis-EMA appears as an interesting alternative due to its reported high DC and low water solubility [14]. The monomer composition is known to influence water sorption and solubility of the final polymer [15,16], which are crucial parameters to determine the longevity of the restorative interface [17]. Thus, varying monomer composition seems to be an alternative for providing a good interaction of luting agents with Y-TZP surface in the long-term.

As regards the photoinitiator systems, classical systems are based on camphorquinone and tertiary amines [10,18]. Camphorquinone is known to be energized by photons of visible light in the range of 400–500 nm, with absorption peak at 468 nm [19], forming an exciplex complex with amine, which generates free radicals responsible for triggering the polymerization reaction [7]. Tertiary amines are essential for the occurrence of polymerization reaction and variations on the type and concentration of these molecules were reported to influence the DC [8,20]. However, some studies have demonstrated enhanced chemical and mechanical properties with the addition of onium salts as a third component in hybrid photoinitiator systems containing camphorquinone and amine [13,21]. Among these photoinitiators, diphenyliodonium hexafluorphosphate (DPIHP) can be added to the conventional CQ/amine system and was reported to provide higher DC [22,23]. These three-component photoinitiator systems generate two initiating free radicals and one initiating cation by a series of electron transfer and proton transfer reactions [24]. Mechanisms suggested for the DPIHP molecule are that carbon-iodine bonds are cleaved by light, reacting with the CQ/amine complex, generating further free radicals that will trigger the polymerization [25,26]. Additionally, aryl sulphonic salts such as triarylsulfonium hexafluoroantimoniate (TAS-Sb) have been also reported to play an important role as a latent catalyst in cationic polymerization, becoming a potential molecule to be added to triple photoinitiator systems [27,28]. However, its application in dental polymers has not been investigated. TAS-Sb is fast curing, soluble in most monomers, such as vinyl ethers and other vinyl monomers, but insoluble in H_2_O [28]. Both DPIHP and TAS-Sb are photoinitiators with different wavelength absorption peaks than CQ, in the UV range, between 220 and 350 nm [29–31]. Hence, further studies evaluating the behavior of adhesive compositions with different monomer mixtures and photoinitiator systems are an alternative to provide better polymerization and superior bonding to Y-TZP.

Another important factor is the light-curing unit (LCU) used during the cementation procedure. Recent polywave LED devices, promise higher DC and polymerization rate for photoinitiator systems that work in variable wavelengths [32]. While the increase in the pulp chamber temperature could occur when using these devices [33] as they present higher irradiance, their use for cementation procedures is interesting since the light attenuation caused by the interposition of ceramics of different opacities could impair the polymerization of resin-based materials [34]. Since DPIHP and TAS-Sb have wavelength absorption peaks in the UV range, it is important to address whether both polymerization and bond strength would benefit from using polywave LCUs. Thus, the aims of the present study were (1) to characterize the physical-chemical properties of experimental adhesives containing monomer mixtures of different viscosities and double and triple photoinitiator systems light activated with monowave or polywave LCUs; and (2) to evaluate their bond strength to Y-TZP when the light attenuation caused by the ceramics interposition is considered. The working hypothesis evaluated was that the adhesive composition (monomer mixtures and photoinitiator systems) and LCUs used in the light-activation would influence the DC, water sorption, water solubility, and bond strength to Y-TZP.

## Materials and methods

### Composition of the experimental adhesives and light-curing units (LCU)

Description of groups with corresponding adhesive compositions is shown in Table 1. Ten experimental adhesives were prepared, including two monomeric mixtures and five photoinitiator systems. The monomeric mixtures were Bis-GMA/TEGDMA and Bis-GMA/Bis-EMA, both at a 1:1 mass ratios. To these mixtures, 0.2 wt% BHT, 10.0 wt% HEMA, and 10.0 wt% ethanol were added. The following photoinitiator systems were then added: CQ/DABE; CQ/DABE/DPIHP; CQ/DABE/TAS-Sb. The light-activation was performed with either a polywave LED device (Valo Cordless, Ultradent Products Inc, South Jordan, UT, USA) or a monowave LED device (DB685, Dabi Atlante, Ribeirão Preto, SP, Brazil). Both LCU devices were used in standard modes, operating at 1000 mW/cm^2^. The irradiance was monitored by positioning the LCU at the same angle, using a laboratory grade radiometer (Demetron, Kerr, Middletown, WI, USA), immediately before the fabrication of each specimen.

**Table 1.** Composition of the experimental adhesives.

Base mixture	Photoinitiator system
Bis-GMA/TEGDMA (1:1) 0.2wt% BHT, 10.0wt% HEMA, 10.0wt% ethanol	0.5wt% CQ, 1.0wt% DABE
	0.5wt% CQ, 1.0wt% DPIHP
	0.5wt% CQ, 0.5wt% DABE, 0.5wt% DPIHP
	0.5wt% CQ, 1.0wt% TAS-Sb
	0.5wt% CQ, 0.5wt% DABE, 0.5wt% TAS-Sb
Bis-GMA/Bis-EMA (1:1) 0.2wt% BHT, 10.0wt% HEMA, 10.0wt% ethanol.	0.5wt% CQ, 1.0wt% DABE
	0.5wt% CQ, 1.0wt% DPIHP
	0.5wt% CQ, 0.5wt% DABE, 0.5wt% DPIHP
	0.5wt% CQ, 1.0wt% TAS-Sb
	0.5wt% CQ, 0.5wt% DABE, 0.5wt% TAS-Sb

Bis-GMA^a^: Bisphenol A glycidyl dimethacrylate; TEGDMA^a^: triethylene glycol dimethacrylate; Bis-EMA^a^: ethoxylated bisphenol A dimethacrylate; BHT^b^: butylated hydroxytoluene; HEMA^a^: 2-hydroxyethyl methacrylate; CQ^b^: camphorquinone; DABE^b^: 1,2 diaminobenzene; DPIHP^b^: diphenyliodonium hexafluorophosphate; TAS-Sb^b^: triarylsulfonium hexafluoroantimoniate.

^a^Sourced from Esstech (Essington, PA, USA); ^b^Sourced from Sigma-Aldrich (St Louis, MO, USA).

### Degree of conversion (DC)

The DC was assessed by analyzing the spectra obtained from an infrared spectrometer (FTIR 8400, Shimadzu Corp., Kioto, Japan) with a resolution of 4 cm^−1^ and 32 scans in the range of 4000–400 cm^−1^. An attenuated total reflection unit (ATR – Miracle ATR, Pike Technologies, Madison, USA) was attached to the spectrometer. The amount of double bonds was determined by adopting absorption peaks corresponding to methacrylate double bonds before and after photoactivation. An initial reading was performed by dropping approximately 12.0 μL of the uncured adhesive to cover the surface of the ATR crystal. A micropipette (Transferpette S, BRAND GMBH + CO KG, Wertheim, Germany) was used to place the amount of adhesive to cover the ATR crystal. After the initial reading, the light-activation was carried out for 10 s with the tip of the LCU as close as possible to the top of the specimen and a second measurement was performed. Five samples per group were evaluated (*n* = 5). The absorption peaks of the aromatic bonds were registered at 1608 cm^−1^ (Abs. 1608) and the peaks of aliphatic double bonds (C=C) were registered at 1636 cm^−1^ (Abs. 1636). The peak of aliphatic bonds decreases with photoactivation, while the peak of aromatic bonds remains stable. Hence, the percentage of remaining double-bonds (%RDB) was determined according to equation below:

(1) $$\%\mathrm{RDB}=(1-(\frac{\frac{\mathrm{Abs}\;1636\text{ cured adhesive}}{\mathrm{Abs}\;1608\text{ cured adhesive})}}{\frac{\mathrm{Abs}\;1636\text{ uncured adhesive}}{\mathrm{Abs}\;1608\text{ uncured adhesive})}}))\;\times \;100.$$

The percentage of DC was then calculated according to equation below:

(2) DC=100−%RDB.

A single operator conducted all DC analyses. Bottles containing each adhesive received codes unknown to the operator conducting the DC evaluation.

### Water sorption (so) and solubility (SL)

For SO and SL another six samples per group (*n* = 6) were prepared by placing the adhesive in a polytetraurethane mold measuring 6 mm in diameter and 1.0 mm in height. The mold was placed over a Mylar strip and a glass slide, and adhesives were poured and light-activated with one of the LCUs previously described (polywave LED device – Valo Cordless or a monowave LED device DB685). Light-activation was performed for 10 s, with the tip of LCU as close as possible to the top of the specimen. Samples were weighed on an analytical balance (Mettler Toledo ML104, Switzerland) until a stable mass was obtained (M0). Samples were then stored in a desiccator containing silica and calcium chloride for 22 h at 37 °C and were further stored in a container with silica for 2 h at 23 °C. After the dehydration cycle, specimens were weighed again using the same equipment until a constant mass was reached (M1). The dimensions (diameter and height) were measured with a digital caliper (Starret, Jiangsu, China) to calculate the volume (V). Subsequently, samples were stored in individual flasks with 10 ml of distilled water and kept at 37 °C for 7 days without changing the liquid. After storage in water, samples were dried with absorbent paper and weighed again (M2). A second cycle of dehydration was conducted as previously described and a final mass was obtained (M3). Finally, SO and SL were calculated according to Eqs. (3) and (4), respectively.

(3) $$\mathrm{SO}\;=\;\frac{\left(M2-M3\right)}{V}$$

(4) $$\mathrm{SL}\;=\;\frac{\left(M1-M3\right)}{V}$$

### Bond strength to Y-TZP

The bond strength evaluation was conducted after the DC analyses. As described in the Results section below, adhesives without tertiary amine (DABE) did not polymerize properly. Thus, only adhesives containing DABE were used in the bond strength evaluation. For this reason, instead of the ten adhesives initially prepared, only six were evaluated (Bis-GMA/TEGDMA with CQ/DABE; Bis-GMA/TEGDMA with CQ/DABE/DPIHP; Bis-GMA/TEGDMA with CQ/DABE/TAS-Sb; Bis-GMA/Bis-EMA with CQ/DABE; Bis-GMA/Bis-EMA with CQ/DABE/DPIHP; and Bis-GMA/Bis-EMA with CQ/DABE/TAS-Sb).

### Preparation of Y-TZP slices

One hundred twenty 1.5-mm-thick zirconia slices (7.5 × 6.0) were prepared for the microshear bond strength (µSBS) evaluation. Slices were obtained by cutting pre-sintered Y-TZP blocks (IPS e.max ZirCAD, Ivoclar Vivadent, AG, Schaan, Liechtenstein) with a sectioning machine (Isomet 1000 Low Speed, Buehler, Lake Bluff, IL, USA) using a diamond disc (Diamond Wafering Blade, Series 15LC diamond n° 11-4254, Buehler, Lake Bluff, IL, USA) at a speed of 275 rpm under constant water-cooling. Slices were washed in tap water to remove cutting debris, fixed in acrylic discs with wax and polished in a metallographic polishing machine (Arotec; Cotia, SP, Brazil) using water cooled silicon-carbide discs (#600, #800, #1200-grade K2000 Polishing Paper, Exakt GmbH, Norderstedt, Germany). Fine-grained felt disk with 1 μm polishing diamond solution (MetaDi water-based suspension, Buehler, Lake Bluff, IL, USA) were used to polish the ceramic slice and a final thickness of approximately 1.2 mm was obtained. After polishing, slices were ultrasonicated with deionized water for 5 min and dried in an incubator at 37 °C for 2 h. Slices were then sintered in a specific oven (Programat S1, Ivoclar Vivadent, AG, Schaan, Liechtenstein) according to program 4 of the manufacturer’s recommendation for fast sintering.

### Surface treatment

All specimens were air abraded with Rocatec Plus (3 M ESPE, Seefeld, Germany) using a sandblaster (Basic Master, Renfert, Germany) at a pressure of 0.28 MPa, distance of 10 mm and angle of 45°, for 15 s. Following air abrasion, Rely X Ceramic primer (3 M ESPE, Seefeld, Germany) was applied on all ceramic slices with a microbrush for 5 s and dried with an oil free air jet. Subsequently, the experimental adhesives were applied with a microbrush and an oil free air jet was applied 10 mm distant from the ceramic surface at an angle of 45° for 5 s. Light-activation was performed for 10 s, according to the adhesive allocated for each group as presented in Table 1.

### Cementation

**Figure 1.**
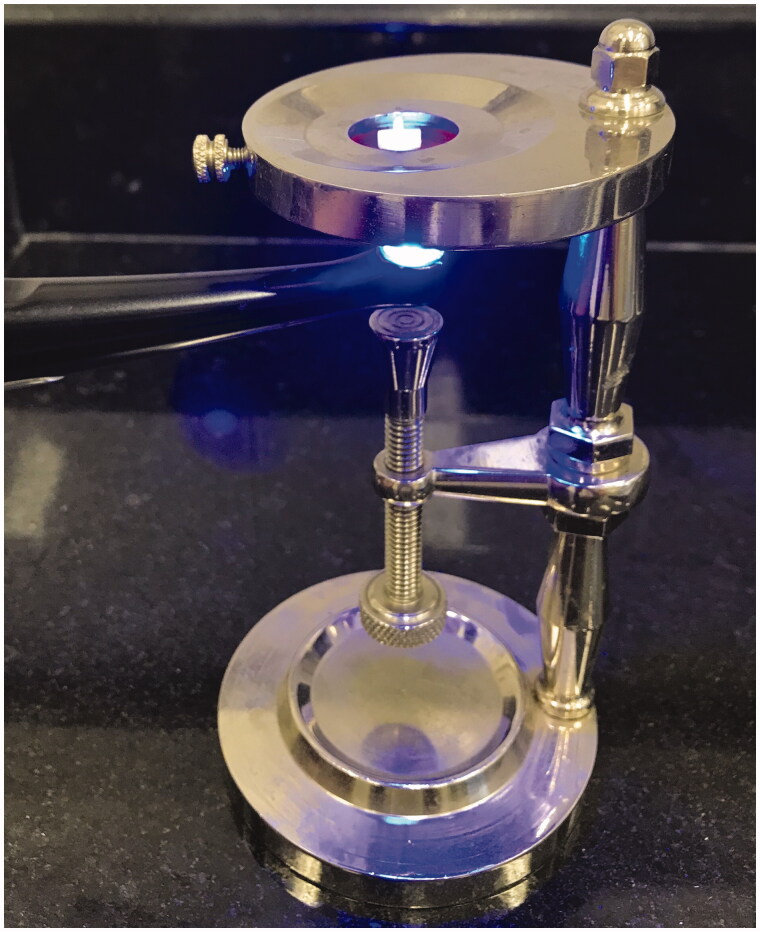
Device used for the cementation procedure.

Prior to cementation, for the purpose of simulating an indirect restoration, resin composite cylinders were made with a resin composite shade A2 (Charisma Diamond, Hereaus Kulzer, Hanau, Germany) using a silicon tube with an internal diameter of 1.4 mm and a height of 1.0 mm. Each cylinder was individually light-activated for 20 s with a Valo Cordless device. Cylinders were then cemented on the treated Y-TZP surfaces as follows: the experimental adhesive (according to the specific group) was applied and light-activated on the upper surface of the composite cylinder, followed by the application of the resin cement (Rely X Ultimate 3 M/ESPE, St. Paul, MN, USA). The resin cement was manipulated according to the manufacturer’s instructions: with the aid of the provided automix syringe, base and catalyst pastes were mixed. After mixing, but before cementation, a small portion of approximately 0.5 mm of the mixed cement was discarded and then the cement was applied to the ceramic surface. The resin cylinder was set on the zirconia surface, which was positioned in an apparatus that allowed light-activation to occur from below the ceramic slice, simulating the attenuation of light during a clinical setting. For this purpose, an apparatus containing an orange filter with a center role of 2 mm in diameter was assembled so the position of the ceramic slice and the LED device underneath could be standardized, ensuring polymerization of the resin cement only through the ceramics (Figure 1). The resin cement was then light-activated for 40 s. The light-activation was performed with either the polywave LED device (Valo Cordless) or the monowave one (DB685), with the tip 3 mm away from the ceramic. Both LCU devices were used in standard modes, operating at 1000 mW/cm^2^.

### Artificial aging

After cementation, samples were stored in distilled water at 37 °C for 24 h. Subsequently, they were thermocycled (model MSCT-3; Elquip Ltda, São Carlos, SP, Brazil) in 5 °C and 55 °C water baths with 30 s of dwell time in each bath for 6000 cycles.

### Microshear bond strength (µSBS)

After aging, samples were subjected to µSBS with a wire-loop method in a universal testing machine (Instron 3342, Illinois Tool Works, Norwood, MA, USA). For that, an orthodontic wire of 0.2 mm was positioned around the base of the resin cylinder, as close as possible to the bonding interface. The measurement of the force applied during the test was performed through a load cell of 500 N and a cross-head speed of 0.5 mm/min. The failure modes were evaluated with a stereoscopic loupe. Failure was assessed as adhesive, cohesive in cement, cohesive in ceramic, or mixed.

### Statistical analysis

DC, SO, SL and µSBS data were analyzed by three-way ANOVA, considering monomer mixture, photoinitiator system, and LCU as independent variables. All analyses were conducted at a level of significance of 5%.

## Results

### Degree of conversion (DC), water sorption (SO), and water solubility (SL)

**Figure 2.**
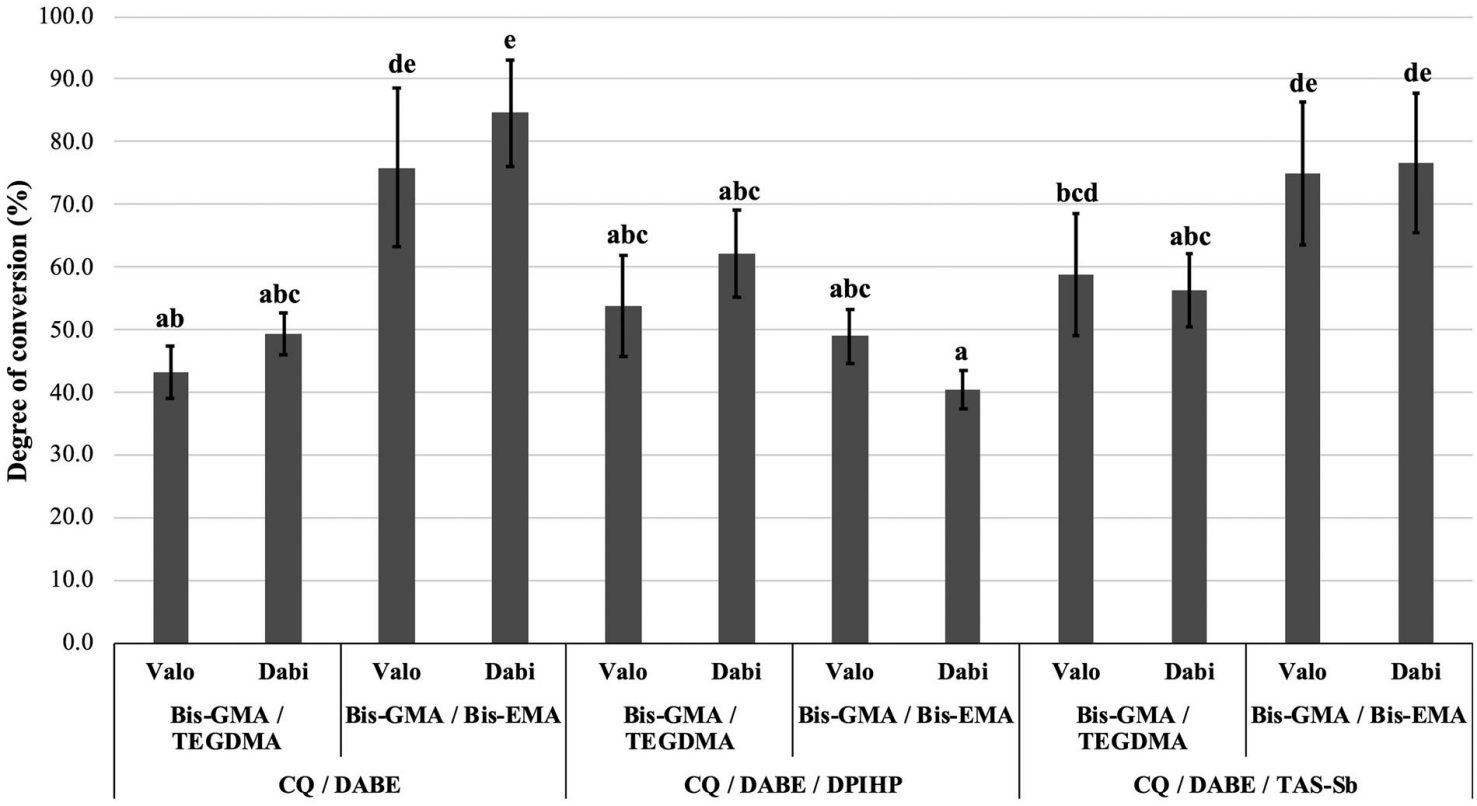
Mean values of degree of conversion (DC) (%). Different letters on top of each column indicate statistically significant difference between experimental adhesives (*p* < .05). Adhesives prepared without DABE did not cure properly and were not included in the analysis.

Mean values and standard deviations of DC, SO, and SL are presented in Table 2. For DC, there were significant differences among monomer mixtures (*p* < .0001) and photoinitiator systems (*p* < .0001). No significant differences were observed between LCUs (*p* = .298). The interaction effect between monomer mixtures and photoinitiator systems was significant (*p* < .0001). Bis-GMA/Bis-EMA showed higher overall DC (*p* < .05). Photoinitiator systems influenced DC in the following order: CQ/DABE/DPIHP < CQ/DABE ≈ CQ/DABE/TAS-Sb. As shown in Figure 2, adhesives containing Bis-GMA and Bis-EMA combined with either CQ/DABE or CQ/DABE/TAS-Sb presented the highest DC, with mean DC higher than 70%. Adhesives containing Bis-GMA/TEGDMA with CQ/DABE and Bis-GMA/Bis-EMA with CQ/DABE/DPIHP presented lowest DC, with values slightly below 50%.

**Table 2.** Mean values and standard deviations of degree of conversion (DC), water sorption (SO), and water solubility (SL).

Monomer mixture	Photoinitiator system	LCU	DC (%)	SO (µg/mm³)	SL (µg/mm³)
Bis-GMA / |TEGDMA	CQ/DABE	Valo	43.3 (4.3)^ab^	56.1 (6.5)^aaaaa^	59.9 (7.3)^daa^
		Dabi	49.4 (3.4)^abc^	58.7 (3.8)^abcaaa^	44.7 (9.8)^bca^
	CQ/DABE/DPIHP	Valo	54.0 (8.1)^abc^	70.9 (1.5)^deaaa^	43.8 (1.3)^bc^
		Dabi	62.2 (6.9)^abc^	69.2 (3.5)^bcdeaa^	44.7 (2.2)^bca^
	CQ/DABE/TAS-Sb	Valo	58.9 (9.8)^bcd^	70.0 (4.98)^deaaa^	24.2 (0.8)^aaa^
		Dabi	56.4 (5.8)^abc^	67.9 (4.8)^bcdea^	26.2 (5.1)^aa^
Bis-GMA / Bis-EMA	CQ/DABE	Valo	76.0 (12.6)^de^	62.1 (2.6)^abcda^	53.4 (6.4)^cda^
		Dabi	84.6 (8.6)^e^	65.5 (3.9)^abcde^	60.1 (11.1)^daa^
	CQ/DABE/DPIHP	Valo	49.0 (4.4)^abc^	58.63 (8.5)^abcaa^	33.5 (1.0)^ab^
		Dabi	40.4 (3.1)^a^	75.2 (9.1)^eaaaa^	31.4 (3.1)^a^
	CQ/DABE/TAS-Sb	Valo	75.0 (11.3)^de^	58.8 (2.7)^abcaaa^	30.11 (7.1)^aa^
		Dabi	76.7 (11.2)^de^	66.8 (3.3)^bcdea^	31.2 (1.7)^a^

For each property, values followed by the same letter are statistically similar (*p* > .05).

**Figure 3.**
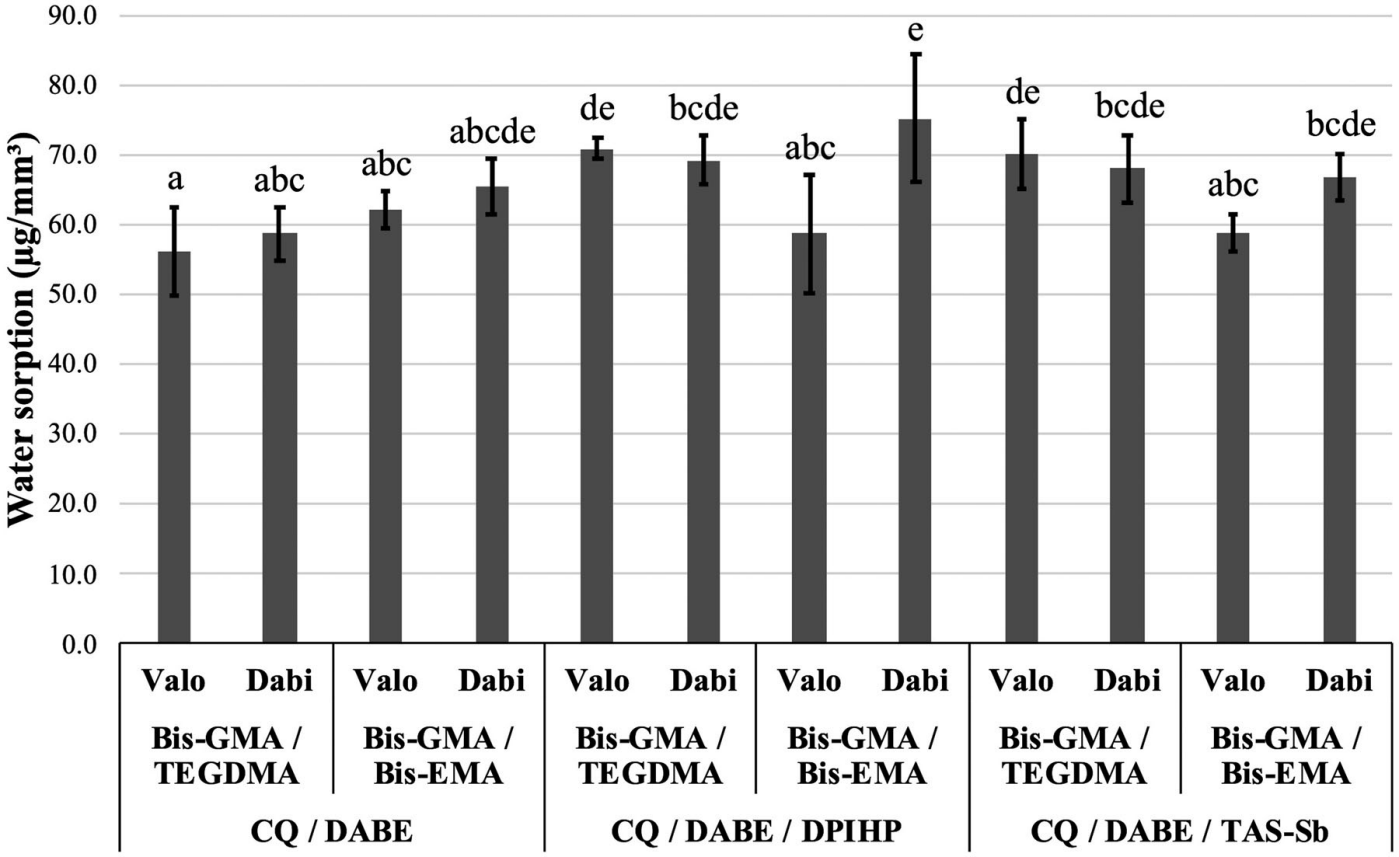
Mean values of water sorption (SO) (µg/mm^3^). Different letters on top of each column indicate statistically significant difference between experimental adhesives (*p* < .05).

For SO, no significant differences were found for monomer mixtures (*p* = .41), while significant differences were found for photoinitiator systems and LCUs (*p* < .001). Significant interactions effects were observed between monomer mixtures and photoinitiator system (*p* < .001); between monomer mixtures and LCUs (*p* < .001); and between monomer mixtures, photoinitiator systems, and LCUs (*p* = .015). Photoinitiator systems influenced SO in the following order: CQ/DABE < CQ/DABE/TAS-Sb ≈ CQ/DABE/DPIHP. The polywave Valo Cordless showed lower overall SO than the monowave Dabi Atlante (*p* < .05). As shown in Figure 3, adhesives containing Bis-GMA/TEGDMA and CQ/DABE cured with the polywave LCU presented the lowest SO values, with a mean value below 60 µg/mm^3^. The adhesive containing Bis-GMA/Bis-EMA with CQ/DABE/DPIHP cured with a monowave device Dabi Atlante, on the other hand, presented the highest SO, with mean value above 70 µg/mm^3^.

**Figure 4.**
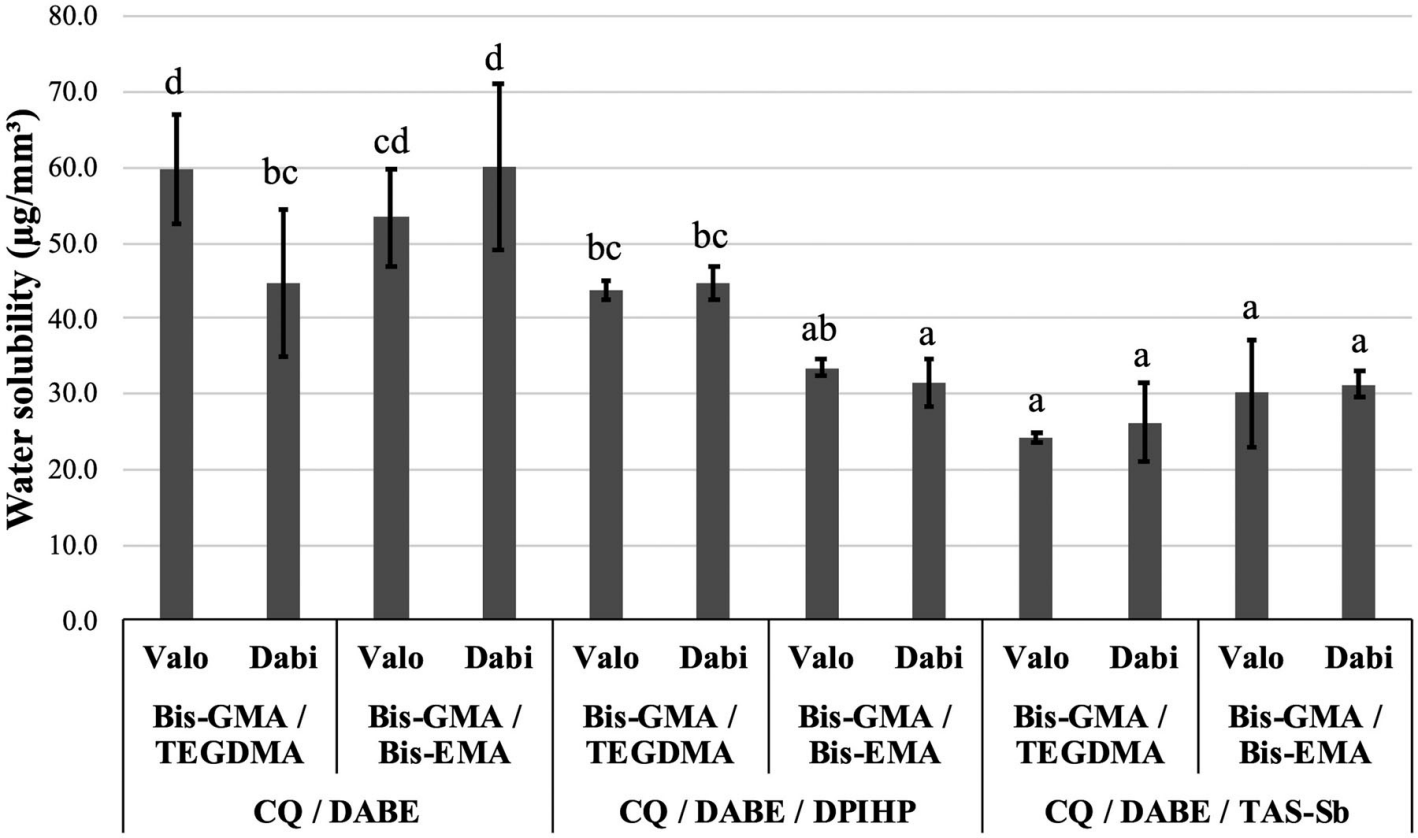
Mean values of water solubility (SL) (µg/mm^3^). Different letters on top of each column indicate statistically significant difference between experimental adhesives (*p* < .05).

For SL, no significant difference for monomer mixture (*p* = .66) and LCU (*p* = .43) was observed, while significant differences were found for photoinitiator systems (*p* < .0001). Significant interaction effects were observed between monomer mixtures and photoinitiator system (*p* < .001); between monomer mixtures and LCUs (*p* = .038); and between monomer mixtures, photoinitiator systems, and LCUs (*p* = .0007). The photoinitiator systems influenced SL in the following order: CQ/DABE/TAS-Sb < CQ/DABE/DPIHP < CQ/DABE. As shown in Figure 4, the lowest mean values of SL were found for adhesives containing Bis-GMA/TEGDMA and CQ/DABE/TAS-Sb light-activated with the polywave LCU Valo Cordless, presenting mean values around 30 µg/mm^3^. Adhesives containing CQ/DABE associated with Bis-GMA/TEGDMA light-activated with the polywave LCU or Bis-GMA/Bis-EMA light-activated with the monowave LCU presented the highest SL, with mean values of 60 µg/mm^3^. It was also observed that all adhesive compositions containing the triple photoinitiator system with TAS-Sb presented lower SL than the conventional CQ/DABE.

### Microshear bond strength (µSBS)

**Figure 5.**
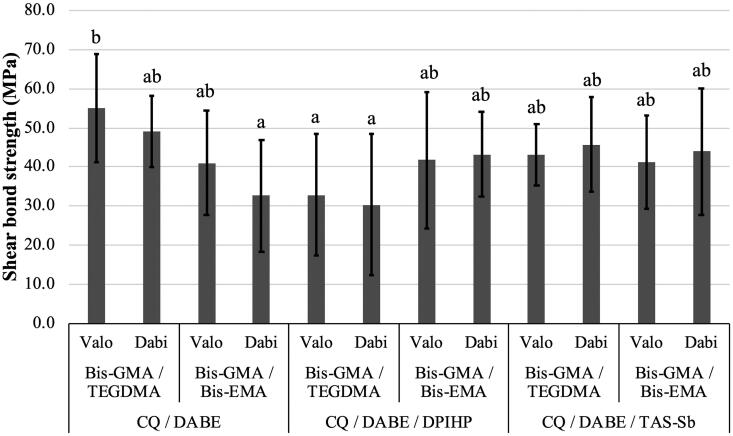
Mean values of shear bond strength (µSBS) (MPa) of experimental adhesives. Different letters on top of each column indicate statistically significant difference between experimental adhesives (*p* < .05).

Mean values and standard deviations of µSBS are presented in Table 3. For µSBS, the only significant factor was photoinitiator systems (*p* = .045). No differences were found for monomer mixtures (*p* = .434) and LCUs (*p* = .516). The interaction effect between monomer mixtures and photoinitiator systems was significant (*p* = .0004). This interaction effect can be observed in Figure 5 which shows that the adhesive composed of BIS-GMA/TEGDMA and CQ/DABE yielded the highest µSBS, presenting mean values around 50 MPa. On the other hand, adhesives containing of BIS-GMA/BIS-EMA and CQ/DABE light-activated with Dabi Atlante, as well as BIS-GMA/TEGDMA and CQ/DABE/DPIHP light-activated with both LCUs presented the lowest µSBS, with most mean values close to 30 MPa. All other experimental adhesives presented intermediate µSBS mean values, which were above 40 MPa, and did not differ significantly between each other or between experimental groups with the highest and the lowest µSBS. Failures were adhesive for all specimens.

**Table 3.** Mean values and standard deviations of microshear bond strength (µSBS).

Monomer mixture	Photoinitiator system	LCU	µSBS (MPa)
Bis-GMA / TEGDMA	CQ/DABE	Valo	55.1 (13.8)^ba^
		Dabi	49.1 (9.0)^aba^
	CQ/DABE/DPIHP	Valo	32.87 (7.85)^aa^
		Dabi	30.4 (18.1)^aa^
	CQ/DABE/TAS-Sb	Valo	43.13 (7.9)^ab^
		Dabi	45.72 (12.1)^ab^
Bis-GMA / Bis-EMA	CQ/DABE	Valo	41.1 (13.1)^aba^
		Dabi	32.6 (14.3)^aa^
	CQ/DABE/DPIHP	Valo	41.8 (17.5)^ab^
		Dabi	43.3 (10.9)^ab^
	CQ/DABE/TAS-Sb	Valo	41.2 (11.9)^ab^
		Dabi	44.0 (16.2)^ab^

Values followed by the same letter are statistically similar (*p* > .05).

## Discussion

This study was designed to evaluate the physical-chemical characteristics and bond strength to Y-TZP of various experimental adhesives light-activated with mono- and polywave LCUs. In the present study, low viscosity monomers such as Bis-EMA and TEGDMA were associated with Bis-GMA. Bis-GMA presents a high molecular weight and hydroxyl groups on its backbone, it is more viscous and shows a compromised polymerization reaction when used alone [8,25]. It was observed that photoinitiator systems were the major factor responsible for differences observed for all properties. Monomer mixture did not influence the overall behavior of all properties, but the three-way ANOVA for DC, SO, SL and µSBS showed that the interaction effects were always statistically significant (*p* < .05). LCUs only influenced SO, but had significant interaction effects on SO and SL. Thus, the working hypothesis was accepted. The present study demonstrated that the addition of Bis-EMA together with the conventional CQ/DABE and triple photoinitiator CQ/DABE/TAS-Sb provided the higher DC in comparison with the addition of TEGDMA to the monomer mixture. Such findings are supported by the studies of Pfeifer et al. and Gajewski et al., who evaluated DC of monomer mixtures containing Bis-EMA [8,9]. Bis-EMA can be considered an analog structure to Bis-GMA due to the presence of ether groups and aromatic rings, differing by the absence of hydroxyl groups that reduce intermolecular forces and viscosity [35]. Both Bis-EMA and TEGDMA have flexible ethylene glycol groups as part of their backbones, which increases conversion compared with Bis-GMA alone [8]. TEGDMA is much more prone to primary cyclization, which also increases conversion, but does not contribute to network formation, and leads to heterogeneity [36]. In previous studies [30,37] Bis-EMA-containing systems led to greater conversion than did TEGDMA, albeit using different initiators at much higher concentrations. In this study, the Bis-EMA containing materials presented the highest DC values, except for the group where DPIHP was used as the iodonium salt in the triple component initiator system.

CQ is a type II free radical diketone photosensitizer molecule which requires a coinitiator, such as a tertiary amine, to form an exciplex complex and produce free radicals that will initiate the polymerization reaction [7,38]. Iodonium and sulfonium salts, such as DHIHP and TAS-Sb, are type I cationic initiators. Hybrid three-component systems can be formulated with a wide variety of photosensitizers in conjunction with the same amine electron donor so the photopolymerizing wavelength may be tailored, including the visible region of the spectrum [24]. In these systems, amines play a major role as highly basic proton scavengers [24,39]. DABE, used in the present study has a relatively high pK_b_ value of 11, and is, therefore, compatible with cationic polymerization [24].

TEGDMA is more hydrophilic than Bis-EMA [40], but the water sorption and solubility are also dependent on the final conversion of the resulting co-polymer with Bis-GMA. The present study demonstrated that water sorption and solubility were dependent on the combination of photoinitiator system and LCU. Solubility showed mean values ranging from 56.1 µg/mm^3^ (for Bis-GMA/TEGDMA – CQ/DABE – Valo Cordless) to 75.2 µg/mm^3^ (for Bis-GMA/Bis-EMA – CQ/DABE/DPIHP – Dabi Atlante), which are similar to the ones obtained in other studies, for both experimental and commercially available adhesives [41,42]. A tendency for most adhesives light-activated with the polywave LCU to present lower SO was observed. Since LCUs had no effect on DC, this result could be explained not only by the wider spectrum, but also by an increase in adhesive temperature caused by the polywave LCU and which could increase the elimination of solvents that might remain in the resin matrix. Furthermore, adhesive formulations of Bis-GMA/TEGDMA with CQ/DABE cured with both LCUs and Bis-GMA/Bis-EMA with triple photoinitiator systems (both DPIHP and TAS-Sb) cured with the polywave device presented the lowest mean values of SO, being, below 60 µg/mm^3^. Hence, the use of polywave LCU is recommended in order to reduce water sorption. SL was significantly influenced by photoinitiator systems, with lower values for CQ/DABE/TAS-Sb. DPIHP also presented lower SL, even though it is considered a hydrophilic molecule due to its iodonium salt [43]. In this sense, all groups containing TAS-Sb in the photoinitiator system differed significantly from all groups containing CQ/DABE, whilst the ones with DPIHP presented intermediate results. Regarding DPIHP, SL was significantly lower for some adhesive compositions as demonstrated in a previous study [44]. The reason for such behavior might be an effect of post-irradiation polymerization [45], attributed to the nature of onium salts that favor the formation of free-radicals due to the regeneration of camphorquinone and decomposition into phenyl reactive radicals [30]. Even though ternary photoinitiator systems showed lower SL, LCUs did not influence this property of the experimental adhesives. Therefore, adhesives containing TAS-Sb in triple photoinitiator systems are expected to present greater resistance to water infiltration at the bonding interface in the long term, independently of the LCU used. This idea, however, should be addressed on future studies.

Since the direct exposure to light only occurs at the restoration margins, most of the activation of the resin cement occurs with the light being transmitted through the ceramics. For this reason, the interposition of ceramics of different microstructures and opacities during cementation procedures is an important issue to be considered in *in vitro* studies as different ceramics affect the polymerization properties of resin-based cements [34,46]. Thus, the present study used a custom-made device to simulate the clinical condition in which the light activation of the resin cement would occur mainly with the light being attenuated by the ceramics. Furthermore, the choice of LCUs with an irradiance of 1000 mW/cm^2^ was based on a previous investigation, which demonstrated that such irradiance applied through ceramic materials showed similar DC in comparison with samples light-activated with 1500 mW/cm^2^ or higher [47].

The µSBS was influenced by photoinitiator systems, corroborating the present working hypothesis. Despite the higher water solubility previously reported for TEGDMA [9] and demonstrated in the present study, the association of this monomer to the CQ/DABE photoinitiator system provided high bond strength even after artificial aging of 6000 thermocycles. However, this adhesive composition did not differ significantly from Bis-GMA/BIS-EMA compositions associated with triple photoinitiator systems containing both DPIHP and TAS-Sb. Thus, these triple photoinitiator systems could be interesting choices for further long-term evaluations considering their lower solubility in water. Although differences between adhesives were found, all mean values of shear bond strength were above 30 MPa and considered higher than reported in previous studies [48,49], a probable explanation being the protocol of sandblasting with Rocatec Plus followed by application of a silane coupling agent previous to cementation. At the same time, it should be noted that 10-methacryloyloxydecyl dihydrogen phosphate (MDP) was not added to the compositions evaluated. Since bonding between polycrystalline ceramics and resin-based materials may be improved by MDP [3,50], these acidic monomers can be considered as further, adjunct adhesion promoters.

The result that LCUs only influenced SO, and not DC, SL, and µSBS, should not be overlooked, especially if a multipurpose adhesive is considered. Since both DPIHP and TAS-Sb showed significantly reduced SL, these photoinitiators could be interesting for further development of adhesives. TAS-Sb as coinitiator of Bis-GMA/Bis-EMA mixtures is of particular interest as, to the best of our knowledge, TAS-Sb has not previously been evaluated as a component of dental adhesives and as it was capable of producing high DC, low SL and bond strengths compared to that of CQ/DABE with mean µSBS above 40 MPa. Lower SL could mean that less unreacted monomers are released into the oral environment. Although it is well-known that all components present in resin-based materials may be released in aqueous solutions [51,52], the cytotoxic potential of leached unreacted monomers is a concern [53].

Some of the limitations of this *in vitro* study should be discussed. The storage medium was water, which was chosen to simplify analysis, since the addition of artificial saliva, for example, would introduce a different set of variables. The storage medium is important as leached out components could be further analyzed. In the SO/SL evaluation samples were stored in individual flasks with 10 ml of distilled water and kept at 37 °C for 7 days. In futures studies, it would be interesting to evaluate the eluted monomers present in the water after this 7-day-water-storage for better understanding of the properties of the evaluated materials. The ceramic surface used here was flat and polished with fine-grained felt disks with 1 μm polishing diamond solution, and not representative of the intaglio surface of a ceramic restoration. This was done to allow for normal distribution of forces during the shear bond strength test. The µSBS evaluation was performed after 6000 thermocycles, which caused expansion/contraction stresses on the resin composite, Y-TPZ and adhesive interface. While previous studies used the same amount of cycles [2,54], longer water storage periods and higher number of thermal cycles could be used in the future to further explore the bonding stability of the designed adhesives. At the same time, thermomechanical aging protocols and chewing simulations should be designed so small samples such as the ones used in the µSBS could be evaluated. The shear test method used is easier and faster than tensile evaluations. At the same time, it allowed a simple modification so the attenuation of light caused by the ceramic interposition could be considered. However, shear bond strength methods have limitations due to the nonhomogeneous stress distribution at the adhesive interface [55], which is significantly affected by the distance between the load application and adhesive interface [56]. During the bond strength evaluation the load applied by the universal testing machine has to be as close as possible to the bonded area, since greater tensile stresses are generated the applied force is farther away from the interface [56]. It should be noted that the wire loop test used in the present study has been shown to have better stress distribution than the knife-edge chisel [57]. Additionally, the µSBS test has a bonding area smaller than 2 mm^2^, which also improves the stress distribution at the bonding interface [56]. The bonding substrate used here was a composite, and not dentin, which was also done to simplify analysis and avoid introducing variables related to the heterogeneity and amount of water present in dentin. While the present study evaluated the bonding to Y-TZP, adhesives were also designed to bond to dentin under moist conditions as HEMA and ethanol were added. Thus, hydrophilic/hydrophobic properties of monomers should be considered, as UDMA, TEGDMA and HEMA are hydrophilic, and Bis-GMA and Bis-EMA are considered hydrophobic [9]. Thus, future studies should consider additional monomer blends, including for example UDMA and HEMA. In moist dentin, adhesives containing the DPIHP and TAS-Sb could offer advantages, since such co-initiators are considered hydrophilic due to their ionic nature, which could favour the photo-activation [13,58]. This is important considering that during bonding procedures to dentin Bis-GMA/HEMA adhesives may present physical phase separation into solid Bis-GMA-rich particles and a fluid-like HEMA-rich phase in the presence of water [59]. In fact, due to the cationic nature of this reaction, systems containing iodonium salt may perform better in the presence of water [13], an important aspect when bonding to the naturally moist dentin.

## Conclusions

LCUs only influenced the water sorption with smaller values being observed for the polywave device. The adhesive containing CQ/DABE/TAS-Sb as coinitiator of Bis-GMA/Bis-EMA mixtures obtained higher degree of conversion and lower solubility. This adhesive also showed bond strength values that were similar to the ones obtained by CQ/DABE.
